# Impact of stirred suspension bioreactor culture on the differentiation of murine embryonic stem cells into cardiomyocytes

**DOI:** 10.1186/1471-2121-12-53

**Published:** 2011-12-14

**Authors:** Mehdi Shafa, Roman Krawetz, Yuan Zhang, Jerome B Rattner, Anna Godollei, Henry J Duff, Derrick E Rancourt

**Affiliations:** 1 Department of Biochemistry and Molecular Biology, Faculty of Medicine, University of Calgary, Calgary, AB, Canada, T2N 4N1; 2 Department of Surgery, Faculty of Medicine, University of Calgary, Calgary, AB, Canada; 3 Department of Cell Biology and Anatomy, Faculty of Medicine, University of Calgary, Calgary, Alberta, Canada; 4 Department of Cardiac Sciences, Faculty of Medicine, University of Calgary, Calgary, AB, Canada

## Abstract

**Background:**

Embryonic stem cells (ESCs) can proliferate endlessly and are able to differentiate into all cell lineages that make up the adult organism. Under particular *in vitro *culture conditions, ESCs can be expanded and induced to differentiate into cardiomyocytes in stirred suspension bioreactors (SSBs). However, in using these systems we must be cognizant of the mechanical forces acting upon the cells. The effect of mechanical forces and shear stress on ESC pluripotency and differentiation has yet to be clarified. The purpose of this study was to investigate the impact of the suspension culture environment on ESC pluripotency during cardiomyocyte differentiation.

**Results:**

Murine D3-MHC-neo^r ^ESCs formed embyroid bodies (EBs) and differentiated into cardiomyocytes over 25 days in static culture and suspension bioreactors. G418 (Geneticin) was used in both systems from day 10 to enrich for cardiomyocytes by eliminating non-resistant, undifferentiated cells. Treatment of EBs with 1 mM ascorbic acid and 0.5% dimethyl sulfoxide from day 3 markedly increased the number of beating EBs, which displayed spontaneous and cadenced contractile beating on day 11 in the bioreactor. Our results showed that the bioreactor differentiated cells displayed the characteristics of fully functional cardiomyocytes. Remarkably, however, our results demonstrated that the bioreactor differentiated ESCs retained their ability to express pluripotency markers, to form ESC-like colonies, and to generate teratomas upon transplantation, whereas the cells differentiated in adherent culture lost these characteristics.

**Conclusions:**

This study demonstrates that although cardiomyocyte differentiation can be achieved in stirred suspension bioreactors, the addition of medium enhancers is not adequate to force complete differentiation as fluid shear forces appear to maintain a subpopulation of cells in a transient pluripotent state. The development of successful ESC differentiation protocols within suspension bioreactors demands a more complete understanding of the impacts of shear forces on the regulation of pluripotency and differentiation in pluripotent stem cells.

## Background

Embryonic stem cells (ESCs) are derived from the inner cell mass (ICM) of pre-implantation embryos [1]. These ESCs have the ability to remain undifferentiated and proliferate indefinitely *in vitro*, while maintaining the potential to differentiate into all three embryonic germ layers [1,2]. An important aspect of ESC research focuses on elucidating the mechanisms of differentiation from the pluripotent ESC to various terminally differentiated cell types. This differentiation capacity makes ESCs an attractive cell source for cell/tissue replacement therapies for the treatment of human degenerative diseases. Moreover, ESCs can also be used as a model system for understanding human genetic disease by elucidating the pathophysiology of specific genetic disorders, including but not limited to cardiac abnormalities.

The *in vitro *differentiation of ESCs into cardiomyocytes provides an opportunity to study the developmental aspects of cardiomyogenesis. Cardiomyocytes are terminally differentiated muscle cells in the adult mammalian heart, which do not divide. Although a small percentage of the cells may be capable of proliferation [3], this is not sufficient for regeneration after myocardial injury. The ultimate goal in cardiac regenerative medicine is to produce in large-scale, highly purified cardiomyocytes which are suitable for cell transplantation. Such cell transplantation therapies would require the successful seeding of as many as 1 × 10^8 ^donor cardiomyocytes per patient [4].

From a commercial perspective, the ability to generate such clinically relevant cell numbers through an economically viable bioprocess is a priority. The robust generation of such large cardiomyocyte numbers could only be feasible in controlled stirred suspension bioreactors capable of maintaining high-density ESC numbers. Currently, most protocols use static culture to differentiate ESCs into cardiomyocytes [5-12]. Although routinely used for ESC culture and differentiation, static culture flasks can only support a pre-clinical research project. Alternatively, stirred suspension bioreactors offer several advantages over the conventional culture methods. We and others have previously demonstrated that suspension bioreactors can support large-scale expansion of the ESCs over extended passages, while retaining their pluripotency [13,14].

The scalable production of ESC- derived cardiomyocytes in a suspension bioreactor system has previously been demonstrated using a retinoic acid based protocol [15,16]. However, since we have previously observed that the suspension bioreactor environment enhances ESC pluripotency, while suppressing differentiation efficiency [17], we sought to investigate whether this phenomenon would also occur in during cardiomyocyte differentiation. This study demonstrates that suspension bioreactor culture systems do indeed have the ability to inhibit differentiation, and even induce 'transient' pluripotency within a defined differentiation protocol, presumably due to influence of shear stress on the cells. Our data shows that in contrast to static culture, ESC cultures induced to differentiate toward cardiomyocytes in suspension bioreactors retain some ability to express pluripotency markers, to form ESC-like colonies, and to generate teratomas upon transplantation.

## Results

### Tumorigenicity of bioreactor-derived cardiomyocytes

**Figure 1:**
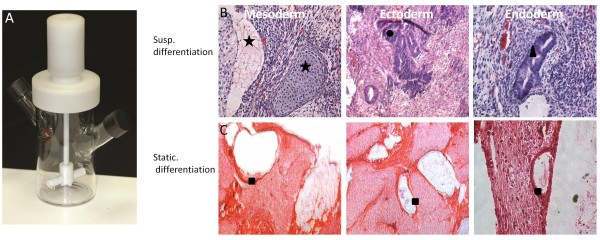
**Pluripotency of bioreactor differentiated cells as shown by tumor formation in SCID mice**. A) The 125-mL NDS stirred-suspension bioreactor. B) Sections of teratoma generated from non-drug selected pluripotent mESCs in bioreactor showing cells from all three germ layers: (Star) Mesoderm; (Circle) Ectoderm; (Triangle) Endoderm (H&E staining). C) Teratoma derived from mESCs after differentiation under static culture system. The majority of teratoma contains unorganized tissue. Only some gut-like epithelial cells were recognizable (Square).

The elegant study by Zandstra *et al*., demonstrated that ESC-derived cardiomyocytes could indeed be generated in a scalable stirred suspension bioreactor provided cells were lineage selected [16]. However, based on our previous observation demonstrating increased tumorigenesis of ESC-derived chondrocytes and osteoblasts in stirred suspension bioreactors, we were interested to determine if this effect was eliminated within the G418 (Geneticin) selected population. Using D3-MHC-neo^r ^ESCs from the Zandstra study, we induced cardiomyocyte differentiation in static and suspension culture for teratoma analysis. The resultant cardiomyocytes from both bioreactor and static culture (with and without G418 selection) were injected into severe combined immunodeficiency (SCID) mice and after 21 days, were harvested and processed for histopathology studies. Interestingly, lineage selected cardiomyocytes from both bioreactor and static differentiation cultures produced no tumor growth *in vivo *suggesting that drug selection for terminal markers may indeed be a reliable tool for the scalable production of ESC derivatives. As expected, static and bioreactor differentiated cells without drug selection during differentiation produced teratomas; however, tumors developed from bioreactor cultured ESCs were significantly larger (~10 mm^2^) than static derived tumors (2-3 mm^2^). Furthermore, teratomas formed from bioreactor ESCs contained cells representative of all three germ layers; whereas, cells from static culture did not. Only some gut-like epithelial cells were recognizable within the teratoma derived from static culture (Figure 1). Based on this result, we undertook the characterization of cardiac bodies generated in the bioreactor, in addition to an analysis of gene expression during differentiation.

### Expression of pluripotency markers and cardiac marker before/after drug selection in suspension and static culture condition

**Figure 2:**
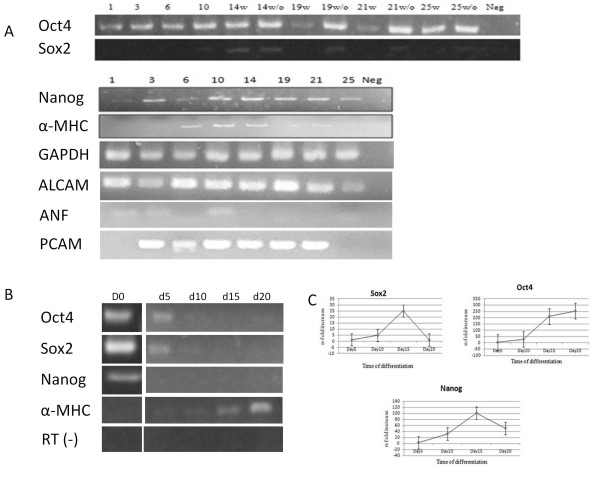
**Expression of pluripotency and cardiac markers during differentiation in suspension culture**. A) RT-PCR showed the expression of pluripotency as well as cardiac lineage genes during differentiation in the suspension bioreactor. The G418 used from day 10 of differentiation to select cardiomyocytes: w: with drug; w/o: without drug, Neg: Negative control. The numbers show days past differentiation. Cardiac markers were examined in non-drug selected bioreactors. B) RT-PCR showed the absence of pluripotency markers gene expression during static differentiation. The expression of the cardiac lineage gene α-MHC increased during differentiation in static culture in non-drug selected cultures. C) Quantitative RT-PCR results in non-drug selected cultures revealed the fold increase of gene expression due to shear forces in suspension bioreactor compared to static culture during the time course of cardic differentiation. Values represent means±SD of 3 independent experiments. p < 0.05.

RT-PCR results confirmed the expression of cardiac genes in addition to the expression of pluripotency markers during differentiation in the suspension culture bioreactor. Cardiac markers were examined in non-drug selected bioreactors. α-MHC (myosin heavy chain) gene expression was up-regulated on day 6 through day 14 in EBs and decreased gradually to the end of the differentiation experiment. The early mesodermal marker PCAM-1(Platelet endothelial cell adhesion molecule-CD31) began to be expressed on day 3 and persisted until day 21. Expression of other cardiogenesis lineage genes such as ALCAM (activated leukocyte cell adhesion molecule, CD166) and ANF (atrial natriuretic factor) showed that the ESCs successfully differentiated to cardiomyocytes in the suspension bioreactor. Among the examined pluripotency markers, Oct4 was consistently expressed on all days during differentiation, surprisingly, even with drug selection present from day 10 of the differentiation protocol. Sox2 expression was absent after G418 selection, but was still expressed in the bioreactor without lineage selection. Nanog expression in the non-drug selected bioreactor fluctuated during the first 10 days of differentiation, with expression being up-regulated at day 14 and decreasing gradually from that time point onward (Figure 2A). In static culture, Nanog expression was down-regulated on day 5 and the pluripotency markers Oct4 and Sox2 were completely absent on day10 after LIF removal (Figure 2B). The cardiac marker α-MHC started to be expressed on day 5 after LIF removal and persisted until the end of experiment. Quantitative RT-PCR in the non-drug selected bioreactors showed that Oct4, Sox2, and Nanog expression persisted during differentiation in the bioreactor culture compared to static culture. The maximum fold increase in expression was on day 15 for Sox2 and Nanog and day 20 for Oct4 (Figure 2C).

**Figure 3:**
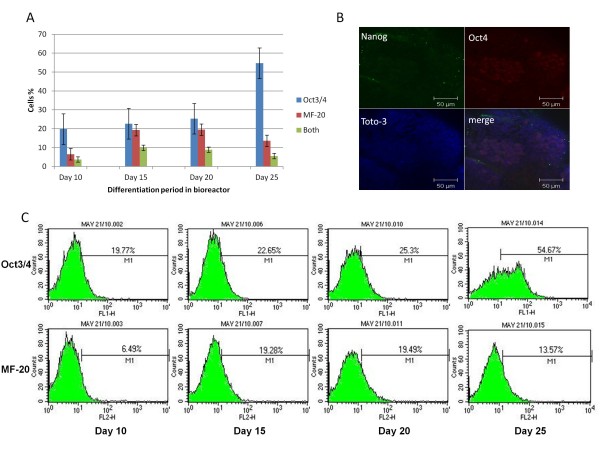
**Expression of pluripotency and cardiac markers after 25 days in suspension culture**. A) Fluorescence-activated cell sorting (FACS) analysis of Oct4 and MF-20. The percentage of cells expressing Oct4 increased from 20% to 54%. The maximum percentage of cells expressing MF-20 was on day 20 and declined afterward. A population of cells was expressing both markers at the same time during differentiation. Statistical analysis (ANOVA) was performed using GraphPad Prism4 (GraphPad Software) and significance was set at p < 0.05. B) Confocal microscopy confirmed the expression of Oct4 and Nanog in a sub-population of cells 20 days after differentiation in bioreactor. C) Distribution of cell populations in suspension culture system as revealed by FACS analysis of Oct4 and MF-20 genes.

**Figure 4:**
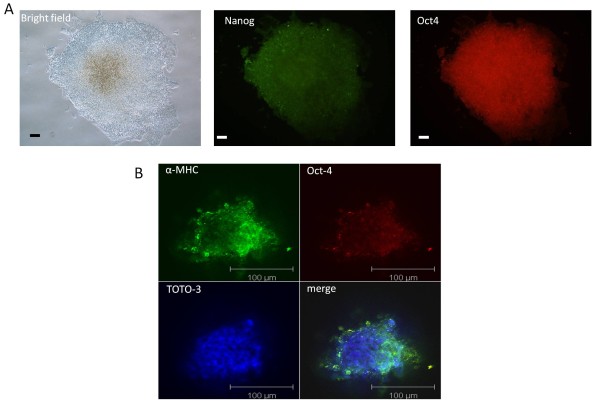
**Pluripotency of bioreactor-differentiated embryoid bodies**. A) Differentiated ESCs retained their ability to form ESC-like colonies upon plating on gelatin-coated culture plates. The morphology of the colonies as well as expression of pluripotency markers showed the pluripotency of differentiated cells after 25 days in suspension bioreactor without drug selection. Immunostaining was performed with antibodies against Nanog and Oct4. B) Confocal microscopy of day 25 EBs showed the simultaneous expression of α-MHC and Oct4 within the bioreactor-derived aggregates. Scale bar: 100 μm.

Since RT-PCR is a global gene expression assay, fluorescence-activated cell sorting (FACS) analysis was used to confirm these marker results at the cellular and protein level in non-drug selected cells. FACS showed that the pluripotency marker Oct4 was expressed during all time points of differentiation examined (Figure 3A, C). In the absence of LIF and G418 selection, the expression of Oct4 positive cells increased from ~20% on day 10 to ~55% on day 25 in the bioreactor. Correspondingly, expression of the cardiac marker, α-MHC (MF-20), increased from ~6.5% on day 10 to ~20% by day 20 with a decrease to ~14% by day 25. Surprisingly, a small population of cells (<10%) expressed both Oct4 and α-MHC (validated with isotype controls) within bioreactors without drug selection. These double positive cells reached a plateau on day 15 during differentiation (Figure 3A). Confocal microscopy also was used to further confirm and validate the existence of Oct4 and Nanog positive cells (Figure 3B). Interestingly, bioreactor-differentiated cells (without drug selection) retained their ability to form ESC-like colonies on gelatin coated plates after 25 days differentiation. However differentiated cells in static culture lost this ability (Figure 4A). Confocal microscopy also confirmed the simultaneous expression of α-MHC and Oct4 in cells within the bioreactor-differentiated EBs (Figure 4B).

### Characterization of bioreactor-derived cardiomyocytes

**Figure 5:**
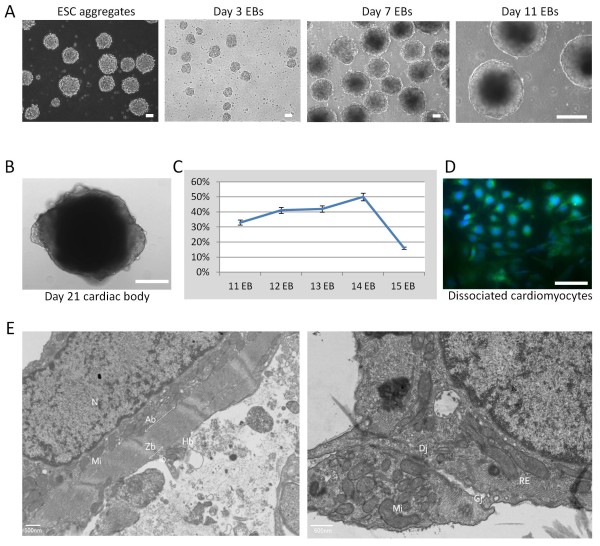
**Mouse ESC^neo ^differentiation in suspension culture**. A) Murine ES cell aggregates. The size of EBs was growing over time period of differentiation. B) Beating cardiac body (CB) 21 days after differentiation. The CBs were beating rhythmically with various rates. C) Percentage of beating EBs. The maximum percentage of beating was 50% on day 14 of EBs. D) Dissociated cardiomyocytes stained with specific cardiac antibody Myosin sarcomere (MF-20) green and DAPI-blue. E) Beating cardiac bodies showed specific subcellular ultrstructure of cardiomyocytes using electron microscopy. The presence the cardiomyocytes specific structure including nucleus and myofibrils, mitochondria, Endoplasmic reticulum, gap junctions and desmosomes was shown. Hb: H-band; Ib: I-Band; Ab: A-band; Zb: Z-band; Dj: Desmosome Junction; GJ: Gap junction; Mi: Mitochondria; RE: Reticulum Endoplasmic; N: Nucleus. Scale bars: 100 μM.

With the observation that pluripotent cells persisted through induced differentiation and the possibility of 'bipotent' Oct4+/α-MHC+ cells, it was next imperative to characterize and test the functionality of the bioreactor derived cardiomyocytes. Within the bioreactor, ESCs aggregated one day after the initial inoculation of single cells without LIF (Figure 5A), with aggregates increasing in size during differentiation. The first beating EBs were seen on day 12, after removal of the LIF and remained for the rest of differentiation period (Figure 5B, Additional file 1). Moreover, the cardiac outgrowths of dissociated aggregates kept beating for almost 45 days with beating rates ranging from 29-60 beats/min. The maximum percentage of beating EBs (50%) was on day 14 after differentiation (Figure 5C). Conversely, the appearance of the first beating EBs in the hanging drop method was on day 6 of EBs, even before moving to suspension culture.

After selection with G418, the resistant derived- cardiomyocytes in the bioreactor had the ultrastructure phenotype of differentiated cardiomyocytes as revealed by light and electron microscope. Dissociated cells were used for light microscope visualization, as well as immunofluorescent staining. After dissociation; the cells produced clusters of spontaneously contractile areas, which were positive for α-MHC as revealed by immunostaining with the MF-20 antibody (Figure 5D). Light microscope showed rounded mononuclear cells with definite subcellular compartments. Transmission electron microscopy of whole cardiac bodies revealed the presence of cardiomyocyte-specific ultrastructure including myofibrils with distinct Z-, I-, H-and A-bands, as well as cell-cell adhesion structures such as gap junctions and desmosomes (Figure 5E).

### Pharmacological responses of ESC-derived cardiomyocytes

**Figure 6:**
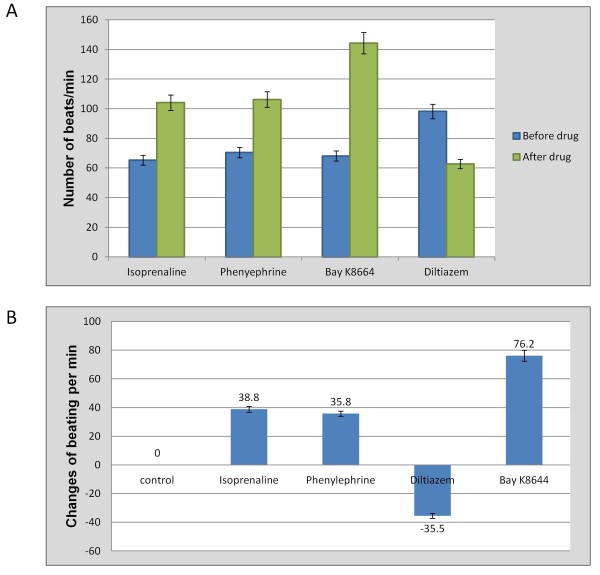
**Chronotropical responses of bioreactor-derived cardiomyocytes**. A) Variation in the number of beatings after drug application. The highest change was due to application of Bay K8664. B) Changes in beating rate. The frequency of changes in beating clearly showed the development of mature ion channels in the membrane of cardiomyocytes, which enabled them to respond to drugs (P < 0.05).

Spontaneously contracting cardiomyocytes were examined to check if they retain the ability to respond to pharmacological agents. Upon administration of Isoprenaline (β1-adrenergic receptor agonist), Phenylephrine (α-adrenergic receptor agonist) and BayK8664 (Ca^2+ ^channel activator), the beating frequency of cardiac bodies were enhanced. However, the chronotropical effect of Diltiazem (Ca^2+ ^channel blocker) treatment was negative on cardiomyocyte beating rate (Figure 6A). The highest increased frequency was observed for BayK8664 which was reversed to the previous beating rate about four hours after removal of the drug through changing the media (Figure 6B). This highlights the existence of developed functional Ca^2+ ^ion channels in cardiomyocytes. These characterization experiments demonstrated that the cells produced were not only positive for cardiac specific markers, but also displayed the properties of functional cardiomyocytes.

## Discussion

The large-scale production of differentiated cells suitable for clinical transplantation is a fundamental objective of regenerative medicine research. The replacement of damaged cells using *in vitro *differentiated functional cardiomyocytes has received more attention in the last decade with clinical trials on the horizon. Despite many developments in the stem cell field, we still lack the ability to produce adequate number of suitable differentiated cells that can meet clinical requirements. We conducted this study to evaluate the tumorgenicity of bioreactor derived cardiomyocytes, since we have observed that ESCs differentiated in stirred suspension culture maintain their pluripotency compare to those differentiated in static culture [17]. In this study we observed that by day 12 following the removal of LIF in suspension, ESCs underwent efficient differentiation into cardiomyocytes in the presence of ascorbic acid and DMSO. Expression of the early mesodermal marker (PCAM) and cardiomyocyte specific markers (ALCAM (CD166) [18], ANF [19], α-MHC and β-MHC [20]) clearly confirmed the differentiation of ESCs into cardiomyocyte in stirred suspension culture. Similarly, in static culture, cells efficiently made EBs three days after the removal of LIF and the derived-cardiac bodies showed characteristics of cardiomyocytes including expression of the molecular marker α-MHC. It has previously been shown that both AA and DMSO enhance differentiation of mouse ESCs into cardiac myocytes. It has been suggested that AA induces permissive changes enabling cardiomyocyte differentiation, while DMSO has been shown to activate essential cardiogenic transcription factors, such as GATA-4 and Nkx-2.5. However, the exact related mechanism for triggering these genes is still not very well known [21,22].

Ultra-structural studies by electron microscopy revealed the specific subcellular sarcomeric organization of cardiomyocytes, such as Z-banding. H- and A-bands, and intercalated discs including desmosome and gap junction were also obvious in derived cells. The chronotropical responses of cardiomyocytes confirmed the existence of specialized Ca^+2 ^channels as well as α1 and β1-adrenergic receptors.

Importantly, no tumors were generated after cardiomyocyte lineage selection used to eliminate cells not expressing the α-MHC-neo^r ^gene. Based on these experiments, we assume that functional cardiomyocytes had been produced that didn't pose a risk of tumor formation *in vivo*. However, it should be emphasized that even in the presence of drug selection, ESCs differentiated in stirred suspension culture, still maintained the expression of pluripotency markers. This trend was even more apparent in cultures not undergoing drug selection pressure, where a sub-population expressed both Oct4 and α-MHC simultaneously in the same cell. It is important to remember, however, that drug selection did negate tumor formation signifying that the expression of pluripotent genes is either: (i) not maintained once the cells are taken out of expansion, or (ii) not sufficient to reprogram a cell that has undergone terminal differentiation.

Furthermore, the results presented here show that even after differentiation toward cardiomyocytes, a sub-population of cells (54%) still express Oct4 demonstrating some link between Oct4 gene expression and the environment. This observation is further confirmed by the difference observed in teratoma formation between static and suspension. The suspension bioreactor differentiations retained cell pluripotency to a degree that ESCs formed teratomas representative of all three germ layers. In contrast, cells from static cultures only formed an unstructured cell population: although not teratomas, tumor masses were still generated. While lineage selection using transgenic constructs is a useful research tool, this approach cannot be applied clinically. Hence, our observation that bioreactor-derived cardiomyocytes maintain hallmarks of pluripotency has significant implications. Before the large scale production of cardiomyocytes in suspension bioreactors will be possible, it will be necessary to eliminate bioreactor induced pluripotency.

We have recently evaluated the application of suspension bioreactor culture for the generation of cartilage and bone tissue [17]. Unlike the static culture environment, bioreactor-differentiated aggregates caused teratoma formation when implanted subcutaneously into SCID mice, which implicated the existence of pluripotent cells in the bioreactor even after 30 days of suspension culture. Upon closer analysis of cells within aggregates, we discovered that cells on the ridges of the aggregate expressed the greatest amount of Oct4 in locations, which would be exposed to the greatest amount of laminar fluid flow. This result suggested that the maintenance of pluripotency within bioreactor differentiation cultures was the result of fluid shear stress. In the current study, much like all our other bioreactor studies, we apply an agitation rate of 100 rpm (shear stress of 6.1 dyne/cm^2^) in order to optimize mass transfer and avoid necrosis, which otherwise occurs at lower velocities [13].

We believe that liquid shear stress in the stirred suspension bioreactor plays an important mechanistic role in bioreactor induced pluripotency. Shear stress can modulate gene expression through mechanotransduction, where physical signals are sensed at the cell periphery, transduced into biochemical signals within the cell, ultimately resulting in cell responses, including changes in gene expression [23,24]. Previous studies in other cells have demonstrated that shear stress can induce the nuclear translocation of ß-catenin into the nucleus [25,26]. As ß-catenin is an important regulator of pluripotency [27], we are interested in the role of the non-canonical Wnt signaling pathway in this induced pluripotency process. Recently, using a LEF/TCF-GFP (Lymphoid enhancer factor/T cell factor) reporter system, we have confirmed that ß-catenin nuclear occupancy is considerably increased over controls when cells are exposed to 6.1 dynes/cm^2 ^shear stress compared to lower levels of shear generated by lower velocities of stirring (unpublished data). Following nuclear translocation, β-catenin forms a complex with LEF/TCF transcription factors. This complex interacts with the specific sequence in the promoter region to activate transcription of certain genes.

Recently, Saha *et al*. has shown that biaxial cyclic strain above a certain threshold inhibits human ESC differentiation and enhances their self-renewal without selecting against growth or survival of differentiated or undifferentiated cells [28]. This group later suggested that strain may induce autocrine or paracrine signaling through TGF-β(Transforming growth factor-beta) superfamily ligands in human ESCs since the TGF-β superfamily activation of Smad2/3 was necessary for suppression of spontaneous differentiation under strain [29].

Despite the fact that stirred suspension cultures are very useful for the generation of a large number of undifferentiated cells, we have found that the addition of medium enhancers is not adequate to force complete differentiation of the population in suspension bioreactors. By elucidating the exact mechanism(s) by which liquid shear stress may contribute to promoting pluripotency and preventing differentiation, we will be able to create an efficient environment for both the production of large quantities of pluripotent stem cells, and their differentiated progeny. This is an important objective for human regenerative medicine, as lineage selection using transgenes will not be possible.

## Conclusions

Although stirred suspension bioreactors are very advantageous for the scalable generation of undifferentiated cells, addition of medium enhancers is not sufficient to induce complete differentiation. Our observation that bioreactor-derived cardiomyocyte cultures maintain characteristics of pluripotency has significant implications for human regenerative medicine. We believe that liquid shear stress in the stirred suspension bioreactor plays an important mechanistic role in bioreactor induced pluripotency, which needs further investigation.

## Methods

### ESC culture

Murine D3-MHC-neo^r ^ESCs (a gift from Dr. Peter Zandstra, University of Toronto) were maintained in the pluripotent state on gelatin-coated tissue culture dishes with inactivated mouse embryonic fibroblasts (MEFs) in high glucose Dulbecco's modified eagles medium (DMEM; Invitrogen) supplemented with 15% FBS (Invitrogen), 0.1 mM non-essential amino acids, 50 U/mL Streptomycin and 50 U/mL Penicillin, 0.1 mM β-mercaptoethanol (Gibco), and 1000 U/mL Leukemia Inhibitory Factor (LIF) (ESGRO, Chemicon). The cells were sub-cultured every second day on MEF feeders. Expansion of pluripotent ESCs in stirred suspension bioreactors was carried out as previously described [13,14].

### Cardiomyocyte differentiation

Differentiation of D3-MHC-neo^r ^ESCs was performed in a 125 ml suspension bioreactor (Corning Style Spinner Flask, NDS Technologies Inc. Figure 1A) by seeding about 5 × 10^4 ^cells/ml into ESC medium without LIF. As an accepted procedure, removal of LIF triggers series of events that cause the spontaneous differentiation of mESCs. Formed embryoid bodies (EBs) were treated with 1 mM ascorbic acid and 0.5% DMSO from day 3 to the end of the experiment to induce cardiomyocyte differentiation. The culture media was changed every 4 days. An agitation rate of 100 rpm was used to achieve the shear stress of 6.1 dyne/cm^2 ^in suspension as described previously [13]. Differentiation on static culture was performed with hanging drop method. Briefly, cell suspensions were diluted to 400-500 cells/20 μl in differentiation medium. Each cell suspension was pipetted onto the inner surface of the tissue culture plate cover and cultured for 3 days. The suspended droplets were transferred into a 100 mm bacteriological plate with differentiation media and incubated for 5 days to form EBs and then plated on gelatin-coated 24-well tissue culture plate for further differentiation. Both ascorbic acid (1 mM) and DMSO (0.5%) were added to induce cardiomyocyte differentiation from day 3 to the end of the differentiation (Day 25). Each differentiation procedure (static or bioreactor) were run as triplicate. Similarly, all of the characterization experiments were performed three times. As the cells carry the MHC-neo^r ^transgene, the expression of the neomycin resistance gene in ESC-derived cardiomyocytes enables their selection using G418 during in vitro differentiation. G418 (400 μg/ml) was added to the cultures from day 10 in order to enrich for cardiomyocytes by eliminating non-resistant, undifferentiated cells. In order to examine the pluripotency of differentiated cells, derived-EBs from suspension bioreactor (without drug selection) were dissociated by trypsin for 5 min followed by plating on gelatin-coated culture plates using mESC media with LIF. Differentiated cells in static culture were also trypsinized for 3 min and replated on gelatin-coated plates using mESC media with LIF (above).

### Teratoma formation assay

CB-17 SCID mice were ordered from Taconic Company and housed in the animal facility of the Faculty of Medicine, University of Calgary. Animal protocols were performed as approved by the University of Calgary Health Science Animal Care Committee. Cells were harvested from both bioreactor and static cultures after three weeks of differentiation using Trypsin/EDTA. Four mice were injected with differentiated cells of each group: 1) bioreactor without G418 selection, 2) bioreactor with G418 selection 3) static differentiation without selection and 4) static differentiation with selection. 10^6 ^cells in a total volume of 100 μl PBS were injected into the skin fold of the each inner thigh. After 21 days, the animals were sacrificed and tissues were dissected and examined by histological procedures. In summary, 4% PFA was used to fix the tissue overnight at 4°C. After dehydration by increasing concentration of ethanol, the tissue was embedded in paraffin. Sections were stained with H&E and examined for different types of tissues by light microscopy.

### Immunofluorescence

Aliquots of ESC aggregates, taken from the bioreactor at various intervals from day 0 to day 25 of differentiation, were washed with PBS, and fixed overnight in 4% PFA in PBS at 4°C. Aggregates were then washed three times with PBS, permeabilized in 0.5% saponin in PBS at 4°C overnight, rinsed three times with PBS, then blocked in 3% BSA at 4°C overnight. The primary antibodies for Nanog, Oct4 and α-MHC (Santa Cruz Biotechnology, Inc. CA, USA) were diluted 1:50 in 3% BSA and added to the aggregates (overnight at 4°C). Approximately 20-25 aggregates were incubated in 50 μl of antibody solution. The aggregates were then washed 3 times with PBS and blocked again overnight at 4°C. Following the blocking step, the aggregates were incubated overnight at 4°C with Alexa-fluor 488 secondary antibody and TOTO-3 (Molecular Probes). TOTO-3, a carbocyanine dimer stain with far-red fluorescence, was used as a nuclear counterstain. After incubation, the aggregates were washed 3 times with PBS and mounted on slides with mountant (9:1 glycerol:PBS). Spacers (0.25 mm) were attached to slides before mounting to avoid aggregate deformation. Slides were analyzed by confocal microscopy (Zeiss 510) using 488, 568 and 633 nm filters. To process the images, LSM image browsing software was used.

For immunofluorescence staining of single cardiomyocytes, EBs were dissociated with collagenase or accutase for 15 min at 37°C. Mechanical dissociation of beating areas was performed under a stereo dissecting microscope using a 27G1/2 needle attached to a 1 ml pipette. Dissociated cells were transferred to fresh media and washed 2-3 times with Dulbecco's Phosphate Buffered Saline (DPBS) and incubated with trypsin for 5-7 min. The cells were plated in 24-well gelatin-coated plates or 35 mm dish for 2 days and then stained with cardiac specific antibody MF-20 (α-MHC).

### Semi-quantitative and real-time PCR

RT-PCR was utilized to evaluate the expression of the pluripotency and cardiac-specific transcripts. RNA was isolated from derived aggregates at various stages of differentiation in static (Day 0, 5, 10, 15, 20) and bioreactor (Day 0, 5, 10, 15, 20 and 25) cultures. The RNeasy Mini Kit (74106, Qiagen) was used to isolate RNA according to the manufacturer's instructions. The RNA concentration was measured by spectrophotometer (BioPhotometer, Ependorf). 1 μg of total RNA was transcribed into cDNA using an oligo dT primer and SuperScript III cDNA synthesis Kit (18080-51, Invitrogen). PCR amplification was carried out in a final volume of 20 μl using Taq DNA polymerase (Invitrogen) using the following steps: 94°C for 3 min, 94°C for 30 sec, 55°C for 45 sec and 72°C for 1 min. Gene primer sets used in the amplification reactions were designed and blasted (NCBI) for mouse ESC specificity. Primer sequences were listed in Table 1. GAPDH was used as an internal standard. PCR products were separated on a 1.5% agarose gel, stained with ethidium bromide, visualized and photographed on a UV trans-illuminator.

**Table 1:** Primer sequences used in RT-PCR and Quantitative-PCR

Gene	Sequences	Product Size	Indication
Oct4	Forward: 5'-GGCGTTCTCTTTGGAAAGGTGTTC-3' Reverse: 5'-CTGGAACCACATCCTTCTCT-3'	312bp	Pluripotency
Sox2	Forward: 5'-CACAACTCGGAGATCAGCAA-3' Reverse: 5'-CTCCGGGAAGCGTGTACTTA-3'	190bp	Pluripotency
Nanog	Forward: 5'-AAGCAGAAGATGCGGACTGT-3' Reverse: 5'-GTGCTGAGCCCTTCTGAATC-3'	232bp	Pluripotency
α-MHC	Forward: 5'-CTGCTGGAGAGGTTATTCCTCG-3' Reverse: 5'-GGAAGAGTGAGCGGCGCATCAAGG-3'	301bp	Cardiomyocyte
ALCAM	Forward:5'-CTTGCACAGCAGAAAACCAA-3' Reverse: 5'-TAGACGACACCAGCAACGAG-3'	190bp	Cardiogenesis
PCAM-1	Forward: 5'-TGCAGGAGTCCTTCTCCACT-3' Reverse: 5'-ACGGTTTGATTCCACTTTGC-3'	245bp	Early mesoderm
ANF	Forward: 5'-TGATAGATGAAGGCAGGAAGCCGC-3' Reverse:5'-GGATTGGAGCCCAGAGTGGACTAGG-3'	203bp	Cardiomyocyte
GAPDH	Forward: 5'-AACTTTGGCATTGTGGAAGG-3' Reverse: 5'-ACACATTGGGGGTAGGAACA-3'	223bp	Housekeeping gene

For quantitative, real-time RT-PCR, standard curves were derived from dilution of the amplicons. Real Time RT-PCR was performed in an iCycler iQ system (Biorad) using a SYBR green PCR master mix (Biorad). The following cycles were used: 3 min at 95°C as initial denaturation, followed by 30 sec at 95°C, 30 sec annealing at 57°C and 30 sec extension at 72°C for 45 cycles. Melting curves were generated at the end of each run to ensure the presence of a single amplicon. Expressions were normalized to GAPDH, a housekeeping gene and compared to static culture. Semi-quantitative and real-time PCR were performed in triplicate.

### Transmission electron microscopy (TEM)

Cells were pelleted, washed in PBS and fixed in 3% glutaraldehyde in Millonig's phosphate buffer for 1 hour at RT. Post-fixation was in 2% OsO_4 _for 20 minutes. The cells were dehydrated in ethanol, and then embedded in Polybed 812 resin (Polysciences, Warrington, PA). Polymerization was performed at 37°C for 24 hours. Silver-gray sections were cut with an ultramicrotome (Leica) equipped with a diamond knife, stained with uranyl acetate and lead citrate, and then examined in a H-7000 Hitachi electron microscope. Fifteen separate samples from suspension bioreactor were used for TEM.

### Chronotropical responses of derived-cardiomyocytes

Beating cardiomyocytes outgrowths were investigated at day 14 of differentiation (4 days after G418 selection). The frequency of beating was calculated by the number of cardiomyocyte pulsations per minute. The beating rate was determined before and after application of each drug. After the addition of drugs, the cardiac bodies were incubated for 5 min before measurement. The variation in beating was measured by the difference of the pulsation rates between pre and post addition of each drug. Twenty separate samples were tested for each drug. The following drugs (all from Sigma) were used: isopernaline (10^-3 ^M, 117H1382-5g), phenylephrine (10^-3^M, P-6126), Diltiazem (10^-3^M, D2521-1g), and Bay K8644 (10^-3 ^M, B-133).

### Flow cytometry

At different time points of differentiation, EBs were harvested from the bioreactor and investigated for expression of pluripotency markers (Oct4, Nanog and Sox2) and cardiac marker α-MHC by flow cytometry. Accutase (eBioscience) treatment for 15 min followed by pipetting was used to dissociate EBs to single cell. Dispersed cells were washed one time with PBS. The pellet was resuspended in 1 ml of 4% paraformaldehyde (PFA) in PBS then washed 3 × 5 min with 4 ml PBS. The cells were permeabilized in 2 ml of 0.5% saponin for 15 min at room temperature. They were washed once with 2 ml PBS for 5 min and resuspended in 3%BSA/PBS for 30 min at 37°C. The primary antibody (approximately 1 μg per 1 million cells) was conjugated with 5 μl of CompA (Invitrogen) and incubated 5 min at room temperature. Then 5 μl of Comp B was added and incubated 5 min. The conjugated primary antibody was then added to cell suspension and incubated for 60 min at 37°C. The cells were washed 3 times with PBS and resuspended in 200 μl FACs tubes. FACs experiments were performed using a BD FACSVantage SE™ System at the University of Calgary's Flow Cytometry Facility.

### Statistical analysis

Results were expressed as means±SD. Significance of quantitative RT-PCR results and chronotropical responses of derived cardiomyocytes was evaluated by the Student's t-test. Difference were considered statistically significant at values of P < 0.05. Statistical analysis (ANOVA) was performed for the FACS results using GraphPad Prism4 (GraphPad Software) and significance was set at p < 0.05.

## Authors' contributions

MS (project design, expansion and differentiation of ESCs, characterization of cardiomyocytes and preparation of manuscript), RK (project design and preparation of manuscript), YZ (differentiation of ESCs), JBR (scanning electron microscope, preparation of manuscript), AG (differentiation of ESCs), HJD (characterization of cardiomyocytes) and DER (project design and preparation of manuscript). All authors read and approved the final manuscript.

## Supplementary Material

Additional file 1 **Beating of bioreactor-derived cardiac body**. The differentiation of mouse ESC into beating cardiac bodies was observed using video microcopy. The first beating EBs were seen on day 12 after LIF removal in stirred suspension bioreactor. Click here for file
